# Appropriate initial antibiotic therapy in hospitalized patients with gram-negative infections: systematic review and meta-analysis

**DOI:** 10.1186/s12879-015-1123-5

**Published:** 2015-09-30

**Authors:** Gowri Raman, Esther Avendano, Samantha Berger, Vandana Menon

**Affiliations:** Center for Clinical Evidence Synthesis, Institute for Clinical Research and Health Policy Studies, Tufts Medical Center, Box 63, 800 Washington Street, Boston, MA 02111 USA; Tufts University School of Medicine, 145 Harrison Avenue, Boston, MA 02111 USA; Tufts University Friedman School of Nutrition Science and Policy, 150 Harrison Avenue, Boston, MA 02111 USA; Currently employed at Baxalta and a former employee of Cubist Pharmaceuticals, 65 Hayden Avenue, Lexington, MA 02421 USA

**Keywords:** Appropriate initial antibiotic therapy, Inappropriate initial antibiotic therapy, Hospital-acquired, Healthcare-associated, Gram-negative, Systematic review

## Abstract

**Background:**

The rapid global spread of multi-resistant bacteria and loss of antibiotic effectiveness increases the risk of initial inappropriate antibiotic therapy (IAT) and poses a serious threat to patient safety. We conducted a systematic review and meta-analysis of published studies to summarize the effect of appropriate antibiotic therapy (AAT) or IAT against gram-negative bacterial infections in the hospital setting.

**Methods:**

MEDLINE, EMBASE, and Cochrane CENTRAL databases were searched until May 2014 to identify English-language studies examining use of AAT or IAT in hospitalized patients with Gram-negative pathogens. Outcomes of interest included mortality, clinical cure, cost, and length of stay. Citations and eligible full-text articles were screened in duplicate. Random effect models meta-analysis was used.

**Results:**

Fifty-seven studies in 60 publications were eligible. AAT was associated with lower risk of mortality (unadjusted summary odds ratio [OR] 0.38, 95 % confidence interval [CI] 0.30-0.47, 39 studies, 5809 patients) and treatment failure (OR 0.22, 95 % CI 0.14–0.35; 3 studies, 283 patients). Conversely, IAT increased risk of mortality (unadjusted summary OR 2.66, 95 % CI 2.12–3.35; 39 studies, 5809 patients). In meta-analyses of adjusted data, AAT was associated with lower risk of mortality (adjusted summary OR 0.43, 95 % CI 0.23–0.83; 6 studies, 1409 patients). Conversely, IAT increased risk of mortality (adjusted summary OR 3.30, 95 % CI 2.42–4.49; 16 studies, 2493 patients). A limited number of studies suggested higher cost and longer hospital stay with IAT. There was considerable heterogeneity in the definition of AAT or IAT, pathogens studied, and outcomes assessed.

**Discussion:**

Using a large set of studies we found that IAT is associated with a number of serious consequences,including an increased risk of hospital mortality. Infections caused by drug-resistant, Gram-negative organisms represent a considerable financial burden to healthcare systems due to the increased costs associated with the resources required to manage the infection, particularly longer hospital stays. However, there were insufficient data that evaluated AAT for the outcome of costs among patients with nosocomialGram-negative infections.

**Conclusions:**

IAT in hospitalized patients with Gram-negative infections is associated with adverse outcomes. Technological advances for rapid diagnostics to facilitate AAT along with antimicrobial stewardship, surveillance, infection control, and prevention is needed.

**Electronic supplementary material:**

The online version of this article (doi:10.1186/s12879-015-1123-5) contains supplementary material, which is available to authorized users.

## Background

In 2011, there were approximately two million cases of hospital-acquired infections in the United States, more than 75,000 of which were fatal [1]. Gram-negative bacteria cause the four most frequent types of hospital-acquired infection: pneumonia, intra-abdominal infection, urinary tract infection (UTI), and bloodstream infection. In the US from 2009 to 2010, 43 % of healthcare-associated infections, 65 % of catheter-associated UTIs, 65 % of pneumonia, and 22 % of central line-associated bloodstream infections were attributed to Gram-negative pathogens [2]. The most important Gram-negative pathogens in the hospital setting include *Escherichia coli*, *Klebsiella pneumoniae,* and *Pseudomonas aeruginosa,* which account for 27 % of all pathogens and 70 % of all Gram-negative pathogens causing healthcare-associated infections [2]. Gram-negative bacteria develop resistance to commonly prescribed antibiotics through mutation and gene acquisition. The incidence of multidrug-resistant, Gram-negative pathogens is on the rise and these organisms represent an urgent threat due to the limited availability of viable therapeutic options [3, 4]

Antibiotic treatment guidelines consistently recommended empiric therapy upon patient presentation with symptoms suggestive of bacterial infection. The potential for resistance must be considered when selecting empiric/initial antibiotic therapy because failure to cover the infectious pathogen (s) is associated with negative outcomes among patients with critical conditions [3]. Although it is well-known that appropriate initial antibiotic therapy (AAT) is associated with favorable outcomes among patients with Gram-negative bacteria, there is a need for an in-depth, comparative analysis of the contemporary literature reporting on outcomes associated with AAT or inappropriate initial antibiotic therapy (IAT). While a number of recent systematic reviews examined the role of resistant pathogens on mortality, as compared with susceptible pathogens, in general, there is a scarcity of information on the role of the timeliness and appropriateness of initial antibiotic therapy in these reviews [5, 6]. In addition, there is considerable lack of information if the effect of AAT as compared with IAT in gram-negative bacterial infections varied by the type of infecting pathogen.

## Methods

We conducted a systematic review and meta-analysis of existing studies on the effectiveness of AAT and IAT for Gram-negative bacterial infections in the hospital setting on clinical and economic outcomes, including cost, length of hospital stay, mortality, and bacterial eradication. This review was conducted according to the Preferred Reporting Items for Systematic Reviews and Meta-Analyses (PRISMA) Statement [7].

### Data sources and study selection

Initial comprehensive literature searches were conducted in MEDLINE, Cochrane CENTRAL, and EMBASE databases from inception through May 2014 for English-language articles published on the use of appropriate or inappropriate empiric/initial antibiotics in patients with hospital-acquired or healthcare-associated Gram-negative bacteria. The searches combined terms for Gram-negative bacteria, appropriate or inappropriate initial antimicrobial therapy, nosocomial or hospital-acquired or healthcare-associated bacterial acquisition, and infections of desired sites such as UTI, intra-abdominal infections, bloodstream infection, and pneumonia. Additional studies were identified by perusing reference lists of systematic reviews and economic reviews or obtained from experts. The results of the literature searches were screened in duplicate using study eligibility criteria; discrepancies were resolved by consensus in group conference. Most publications identified by our initial search examined the effect of use of AAT or IAT on mortality. Therefore, searches were expanded to include community-acquired Gram-negative infections to identify additional articles relevant to economic outcomes of length of stay or cost.

#### Study inclusion criteria

We included studies of adult patients with susceptible, resistant, or multidrug-resistant Gram-negative infections of the following sites: respiratory, intra-abdominal, bloodstream, and urinary tract. While studies with nosocomial, hospital-acquired, or healthcare-associated infections were included for all outcomes, studies with community-acquired Gram-negative infections were included only for the outcomes of length of stay and cost. Patients had to have been given empiric antibiotic therapy prior to the identification of culture results. Individual study definitions of AAT or IAT were accepted. Additional study inclusion criteria included sample size of at least 10 patients per comparison group (AAT versus IAT) evaluating at least one of the following outcomes: mortality, clinical success, microbiologic eradication, length of stay (hospital and intensive care unit [ICU]), or cost. For studies with multiple publications on the same Gram-negative organism, we included those with the longest recruitment period or longest follow-up, largest sample size, or both. Unpublished literature was not included, and no authors were directly contacted for unpublished data.

#### Study exclusion criteria

We excluded narrative reviews, cross-sectional studies, case reports, editorials, letters, comments, and non–English-language articles. Studies that included patients with Gram-positive bacteria, fungi, or polymicrobial infection that did not stratify results by Gram-negative bacteria were excluded. Studies where all patients (100 %) received either AAT or IAT were excluded.

### Data extraction and quality assessment

Each article was screened by one of three investigators and confirmed by at least one other. Included studies were then extracted independently, and results were confirmed by one of the other investigators. The extracted data included study design; participant characteristics; comorbidity score; comorbidities; site of infection; primary cause of infection; history of antibiotic use; history of hospitalization; inclusion criteria; exclusion criteria; definitions of AAT or IAT; percentage of patients receiving AAT; percentage of patients receiving IAT; and unadjusted or adjusted analyses comparing outcomes of interest in patients who received AAT or IAT.

### Data synthesis and analysis

We considered the following outcomes for inclusion in the meta-analysis: all-cause mortality in hospital, infection-related mortality, length of stay, hospital costs (as defined by study authors as direct and indirect costs incurred during an inpatient stay, or as hospital accounting costs), and clinical cure or microbiological clearance. Meta-analysis was conducted using the random effects model; results are reported as summary odds ratio (OR) [8]. The random effects meta-analyses assessed any potential differential impact of AAT or IAT on the outcomes of interest using unadjusted and adjusted data, when feasible. The proportion of IAT and the proportion of AAT add up to 100 % for unadjusted mortality data and results data were rounded to two decimal places. Cochran’s Q chi-square test was used to test for between-study heterogeneity and quantified with I^2^ [9]. Additional subgroup analyses were conducted by outcome time points, site of infection, pathogen and definition of AAT. For meta-analyses with at least 10 studies, we evaluated the potential for publication bias with funnel plots and Egger’s tests for small study effects [10]. We looked for differences across studies using stratified analyses to explain heterogeneity in association results. To assess study quality, we applied quality questions from the Newcastle–Ottawa Quality Assessment Scale for case-control and observational studies [11]. When feasible, sensitivity analyses were conducted by excluding studies that were rated as having high risk of bias. All analyses were performed in Stata version 13 (StataCorp, College Station, Texas).

This review evaluated data from published studies and was exempt from ethics committee approval. This review did not involve any direct research on patients, and no informed consent was required.

## Results

**Fig. 1:**
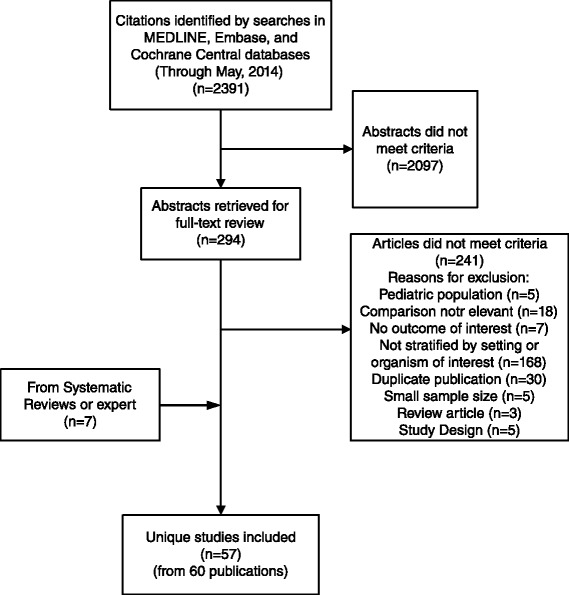
PRISMA flow diagram

The literature search identified a total of 2391 abstracts, of which we screened 294 in full text and added seven articles from existing systematic reviews and by experts. A total of 57 studies in 60 publications were included (Fig. 1). In addition, the figure includes the reasons why the 241 full-text articles were excluded.

Appropriate use of antibiotic therapy was defined heterogeneously among 68 % of included studies using both susceptibility and timeliness. About 20 % of studies reported susceptibility to at least one empiric antibiotic therapy by subsequent culture examination as the definition of AAT. The administration of empiric antibiotics within a specified number of hours of index infection site culture was reported as the only definition in 8 % of studies. The specified number of hours varied between 24 and 72 h. There were no definitions reported for AAT in 4 % of studies.

Only one-half of the eligible studies adjusted for confounders in their analyses for outcomes of interest. Seven studies (12 %) included only resistant pathogens and the remaining studies included both resistant and susceptible pathogens. Patients with resistant pathogens differed in sex distribution, associated co-morbid conditions, had central venous catheter or urinary catheter, or had an ICU stay, as compared with those with susceptible pathogens.

Of the five outcomes examined, mortality was the only endpoint for which a meta-analysis was feasible. For the other four outcomes, a meta-analysis was not possible due to insufficient data or heterogeneity of data. For the mortality analysis, we were able to stratify by reported outcome time point, the type of pathogen, definition of AAT, and ICU-related infection.

### Mortality outcomes

Thirty-nine studies in 41 publications representing a total of 5809 patients examined the outcome of mortality [12–53] (see Additional file 1: Table S1). Of these, five were prospective observational studies and 34 were retrospective (see Additional file 2: Table S2). Most retrospective studies identified eligible patients from hospital administration databases, and four studies examined patients admitted to the ICU [12, 18, 27, 48]. All studies were conducted in academic hospitals or tertiary care centers and most were single-center studies, except for two multicenter studies [42, 47].

Included studies were conducted in the US (five studies), Europe (17 studies), Asia (14 studies), and South America (three studies). The average age of patients among included studies ranged between 41 and 76 years. The proportion of men included in studies ranged from 49 to 81 %. The majority of infections examined were bloodstream infections; only five studies evaluated patients with hospital-acquired pneumonia. Mortality was reported within 14 days (12 studies) and 30 days (22 studies) with two studies reporting at both time points [19, 43]; the mortality time point was not documented in seven studies. Only four studies reported data on infection-related mortality [14, 22, 24, 53].

Twenty-nine studies (72.5 %) reported any baseline comorbidity score, heterogeneously, with higher baseline scores among non-survivors (see Additional file 1: Table S1). Coexisting conditions among included study populations were diabetes (0.6 to 40 %), immunosuppression (3.5 to 100 %), kidney disease (4 to 37.6 %), cardiovascular diseases (5 to 81 %), cerebrovascular diseases (0.8 to 50 %), chronic obstructive pulmonary diseases (7.9 to 31 %), cancers (7.5 to 100 %), hypertension (22.1 to 52.6 %), artery disease (10 to 17.2 %), liver disease/cirrhosis (1.9 to 15.4 %), and lung disease/dysfunction (9 to 17.4 %).

**Fig. 2:**
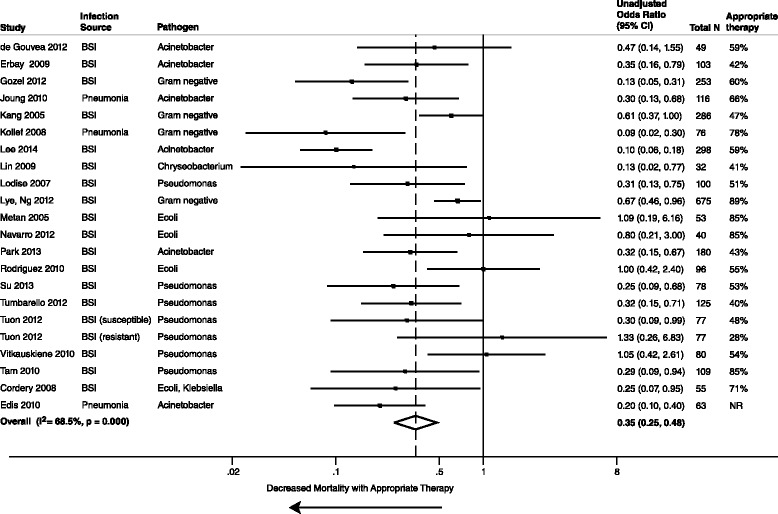
Whisker plot of unadjusted mortality at 30 days among patients receiving AAT

**Fig. 3:**
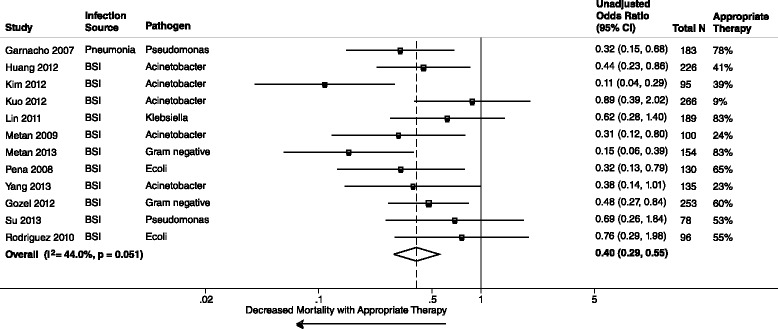
Whisker plot of unadjusted mortality at 14 days among patients receiving AAT

**Fig. 4:**
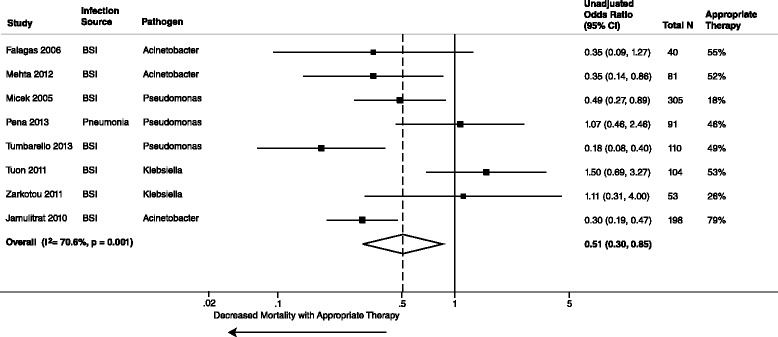
Whisker plot of unadjusted in-hospital mortality among patients receiving AAT

All 39 studies reported unadjusted data on the association of AAT and mortality. The meta-analysis of unadjusted data demonstrated a statistically significant decreased mortality in those who received AAT compared to those who received IAT (39 studies, 5809 patients, unadjusted summary OR 0.38, 95 % CI 0.30–0.47; I^2^ = 64.9 %). Stratified analyses by mortality time point concurred with the overall unadjusted mortality (Figs. 2, 3 and 4). Additional stratified meta-analyses of studies that defined the use of AAT by timeliness of index culture and studies that examined extended-spectrum β-lactamase *E. coli* and *Klebsiella spp* were homogeneous (Table 1). Conversely, IAT increased risk of mortality (unadjusted summary OR 2.66, 95 % CI 2.12–3.35; 39 studies, 5809 patients).

**Table 1:** Subgroup analyses of unadjusted mortality in hospitalized patients receiving AAT

Characteristics	Subgroups	N Studies	Unadjusted OR	Results: 95 % CI	*I* ^*2*^	Cochran Q
						*p*-value
Main analysis	All patients	39	0.38	0.30, 0.47	*64.9 %*	<0.001
Non-Acinetobacter	Excluding Acinetobacter spp studies	24	0.46	0.34, 0.60	*61.4 %*	<0.001
Mortality outcome time point	14-day	12	0.40	0.29, 0.55	*44 %*	0.05
	30-day	21	0.35	0.25, 0.48	*68.5 %*	<0.001
	In-hospital (time NR)	8	0.51	0.30, 0.85	*70.6 %*	0.001
Pathogen	Acinetobacter	15	0.29	0.21, 0.39	*51.9 %*	0.01
	Gram-negative	6	0.27	0.13, 0.53	*81.1 %*	<0.001
	ESBL *E. coli*	4	0.66	0.35, 1.22	*18.7 %*	0.30
	*Klebsiella*	3	1.00	0.57, 1.78	*16.6 %*	0.30
	*pseudomonas*	11	0.41	0.29, 0.60	*45.0 %*	0.05
Pathogen and source of infection	ESBL BSI	6	0.71	0.38, 1.31	*48.1 %*	0.086
	*Klebsiella* BSI	2	0.74	0.37, 1.46	*0.0 %*	0.453
	*A Pneumonia*	2	0.24	0.14, 0.40	*0.0 %*	0.466
	*A* BSI	13	0.30	0.21, 0.43	*57.4 %*	0.005
	*P.* pneumonia	2	0.58	0.18, 1.89	*77.4 %*	0.036
	*P* BSI	9	0.38	0.26, 0.56	*35.6 %*	0.133
	*Gram-negative* BSI	4	0.33	0.16, 0.70	*83.7 %*	<.0001
	*Gram-negative* pneumonia	1	0.09	0.03, 0.30	*NA*	NA
ICU-related infections by included subjects	≤50%	12	0.413	0.30, 0.57	*40.5 %*	<0.001
	>50%	23	0.38	0.27, 0.53	*68.9 %*	0.07
	Not reported	4	0.27	0.11, 0.67	*83.2 %*	<0.001
AAT timeliness with regard to initial culture	≤24 h	11	0.50	0.35, 0.71	*50.1 %*	0.024
	≤48 h	9	0.40	0.22, 0.72	*78.8 %*	<0.001
	≤72 h	7	0.29	0.22, 0.39	*0.0 %*	0.73
	Timeliness NR	10	0.32	0.20, 0.52	*66.2 %*	0.001
Definitions of AAT	Timeliness and susceptibility	26	0.37	0.28, 0.49	*68.3 %*	<0.001
	Timeliness alone	3	0.56	0.25, 1.23	*35.4 %*	0.213
	Susceptibility alone	8	0.36	0.23, 0.55	*50.0 %*	0.051
	Not reported	2	0.31	0.03, 3.61	*90.1 %*	0.001

*A* Acinetobacter, *AAT* Appropriate initial antibacterial therapy, *BSI* Blood stream infection, *CI* Confidence Interval, *E.coli* Escherichia coli, *ESBL* Extended spectrum beta-lactamase, *hr* hour, *ICU* Intensive care unit, *OR* Odds ratio, *P* Pseudomonas, *N* Number, *NR* Not reported

**Fig. 5:**
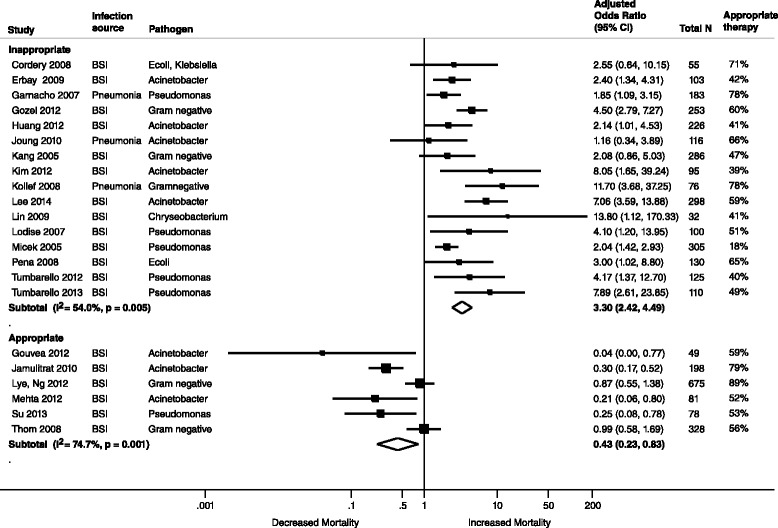
Whisker plot of adjusted mortality among patients receiving AAT or IAT

Twenty-two of 39 studies reported data for an adjusted meta-analysis (Fig. 5). Adjusted data from 16 studies (2493 patients) demonstrated increased mortality with IAT (adjusted summary OR 3.30, 95 % CI 2.42–4.49, I^2^ 54 %) or decreased mortality in 6 studies (1409 patients) with AAT (adjusted summary OR 0.43, 95 % CI 0.23–0.83, I^2^ 75 %).

Funnel plots of all studies reporting unadjusted and adjusted mortality with IAT indicated no potential for missing studies with inverse associations (RR <1.0).

#### Economic outcomes

Seventeen studies in 19 publications representing a total of 3855 patients examined the economic outcomes of cost or length of stay [17, 22, 25, 31, 38, 46, 54–66]. None were prospective, and all 17 were retrospective observational studies (see Additional file 3 Tables S3 and S4). Included studies were conducted in the US (six studies), Europe (four studies), Asia (six studies), and Israel (one study). The majority of infection was bloodstream infections (10 studies), and the remainder were UTIs (three studies), pneumonia (two studies), and intra-abdominal infections (two studies).

Of the 10 studies that examined cost outcomes, four provided direct evidence of an association between IAT and cost, the remaining six provided indirect evidence for an association. In four studies with the direct evidence of an association between IAT and cost, IAT was associated with higher mean total cost (two studies), hospital costs (two studies), or antibiotic cost (one study). In six studies with indirect evidence, patients with resistant organisms received more IAT than those with susceptible organisms, and the resistant groups had higher mean total cost (two studies), hospital costs (two studies), antibiotic cost (one study), or per-patient cost (one study) compared with the susceptible group.

Heterogeneity of outcomes precluded a meta-analysis and only a small number of studies reported quantitative data for direct evidence. IAT was associated with longer hospital stay (five studies), but shorter ICU stay (two studies). Receiving IAT was associated with higher hospital cost (two studies; median = $51,977; interquartile range [IQR] $34,644–$69,311) and longer hospital stay (five studies; median = 21; IQR 13–21 days) as compared with AAT (median = $40,187; IQR $25,982–$54,392; median = 18; IQR 9–24 days, respectively).

#### Clinical response outcomes

Five studies reported data on clinical response including microbiological clearance (one study) and treatment failure (four studies) [17, 67–70]. The lone study on microbiological clearance identified that IAT was significantly associated with a slower initial rate of bacterial clearance, as compared with AAT [67]. All four studies in unadjusted analyses and one study in adjusted analysis found statistically significant decreased treatment failure with AAT, as compared with IAT [17, 68–70]. The meta-analysis of unadjusted data demonstrated a statistically significant decreased treatment failure with AAT (three studies, 283 patients, OR 0.22, 95 % CI 0.14–0.35), as compared with IAT. Only one study that measured treatment failure defined as persistence of the presenting signs of infection 72 h after initial culture collection [68].

## Discussion

This systematic review of the literature demonstrates the impact of the use of AAT or IAT on clinical and economic outcomes among patients hospitalized with Gram-negative infections. A meta-analysis of studies examining the impact of use of AAT in hospital-acquired, Gram-negative infection indicates a significant decrease in risk for mortality compared with the use of IAT. Hospital and ICU length of stay was prolonged with IAT compared with AAT. There were insufficient data that evaluated AAT for the outcome of costs among patients with nosocomial infections.

The discovery and development of antibiotics is one of the major health advances of the 20th century; however, the global spread of antimicrobial resistance is rendering many commonly-used antibiotics obsolete [71]. The development of resistance occurs naturally over time, but is accelerated through the overuse and/or misuse of antibacterial therapies [72–74]. The rapid global spread of resistant bacteria and subsequent loss of antibiotic effectiveness increases the risk of IAT in two ways: first, patients with resistant infections may initially be given a drug with little or no activity due to resistance, effectively delaying the time to treating the pathogen and, second, the need to treat resistant infections may lead to antibiotics with proven activity being used too widely and too early, further promoting the spread of resistance and perpetuating the cycle. Guidelines recommend empiric therapy coverage against anticipated pathogen and resistance that should be started upon patient presentation with symptoms suggestive of bacterial infection. This should be followed by de-escalation therapy once the pathogen and sensitivities are known.

IAT is associated with a number of serious consequences, including an increased risk of hospital mortality. A US single-center, retrospective cohort study assessing the role of multidrug resistance in patients with Gram-negative sepsis and septic shock found that a higher proportion of patients who had a multidrug-resistant infection or received IAT died [75]. Non-survivors were three times more likely to receive IAT than those patients who survived their hospitalization (43.4 % vs. 14.6 %) highlighting the fact that IAT is a key predictor of mortality in patients with serious Gram-negative infections [75].

Infections caused by drug-resistant, Gram-negative organisms represent a considerable financial burden to healthcare systems due to the increased costs associated with the resources required to manage the infection, particularly longer hospital stays. In 2009, the European Centre for Disease Prevention and Control and the European Medicines Agency estimated that the total healthcare cost incurred in Europe due to resistant infections totaled at least €1.5 billion each year, with the main portion accounted for by resistant Gram-negative pathogens at €867 million. These costs include additional in-hospital and outpatient care costs and the societal costs of productivity losses due to absence from work and death [76]. For the US, estimates are as high as US$20 billion in extra direct healthcare costs, with additional costs to society for lost productivity as high as US$35 billion per year [77].

This review and meta-analysis results should be interpreted in light of its limitations. Our inclusion criteria required that studies only consider hospital-acquired or healthcare-associated infection and were published in English for the outcome of mortality. For the evaluation of economic outcomes, we expanded the eligibility to community-acquired infection given the paucity of data on economic outcomes. In addition, the limitations of this review reflect, to a large extent, the limitations of the data available in primary studies. There was a general lack of adequate accounting for possible confounders, with little over one-half of the studies (59 %) reporting adjusted analyses for mortality outcomes. Confounding factors may have influenced the outcome of mortality in unadjusted analyses. Although the proportion of IAT and the proportion of AAT add up to 100 % for unadjusted mortality data, both data are presented for ease of comparison. In contrast, all studies did not report adjusted data for both groups. Finally, the literature is heterogeneous with respect to the definition of use of IAT. As a result, we performed subgroup analyses based on different definitions reported in studies. Given the nature of reporting and data collection for the studies included in this meta-analysis, we were unable to examine culture negative cases or other definitions of appropriate therapy such as guideline-concordant. There were fewer studies that evaluated the outcome of costs or length of stay for nosocomial infections. These data are likely an underestimate of the true economic impact of IAT as they do not account for reduced resource utilization and shorter hospital stay attributable to the higher mortality in patients receiving IAT. Analyzing the impact of resistance on length of stay and costs is difficult due to competing events of mortality and discharge or time-dependent bias, which were not appropriately addressed in most of the included studies.

## Conclusions

Resistance rates are increasing among Gram-negative pathogens that are responsible for serious nosocomial infections, including *P. aeruginosa* and extended spectrum β-lactamase–producing *Enterobacteriaceae.* Our study presents a review of contemporaneous literature and demonstrates that IAT is associated with increased mortality and prolonged hospital stays that could translate into higher health care costs. Conversely, AAT improves patient outcomes and could potentially lead to cost savings. These findings underscore the need for technological advances for rapid diagnostics as well as the “treat the right patient with the right drug at the right time” approach to treating serious nosocomial infections, particularly when there is a high clinical suspicion of resistance. A global multidisciplinary effort to combat resistance that includes antimicrobial stewardship, infection control and prevention, and the development of new antimicrobial agents with activity against multidrug-resistant Gram-negative pathogens is critical to combat this public health threat and prolong the effectiveness of existing antibiotics.

## Additional files

Additional file 1: Table S1. Characteristics of included studies reporting mortality outcomes. (DOCX 98 kb) Additional file 2: Table S2. Additional characteristics of included studies reporting mortality outcomes. (DOCX 95 kb) Additional file 3: Table S3. Study characteristics and results of economic outcomes. **Table S4.** Study characteristics and results of length of stay (DOCX 74 kb)

## Abbreviations

AAT: Appropriate antibiotic therapy
CI: Confidence interval
IAT: Inappropriate antibiotic therapy
ICU: Intensive care unit
IQR: Interquartile range
OR: Odds ratio
PRISMA: Preferred reporting items for systematic reviews and meta-analyses
UTI: Urinary tract infection

## References

[1] Healthcare-associated Infections (HAIs). HAI Prevalence Data and Statistics. Centers for Disease Control and Prevention website. Available online: http://www.cdc.gov/HAI/surveillance/index.html. Accessed January 12, 2015

[2] Sievert DM, Ricks P, Edwards JR, Schneider A, Patel J, Srinivasan A (2013). Antimicrobial-resistant pathogens associated with healthcare-associated infections: summary of data reported to the national healthcare safety network at the centers for disease control and prevention, 2009–2010. Infect Control Hosp Epidemiol.

[3] Boyd N, Nailor MD (2011). Combination antibiotic therapy for empiric and definitive treatment of gram-negative infections: insights from the society of infectious diseases pharmacists. Pharmacotherapy.

[4] Livermore DM, Woodford N (2006). The beta-lactamase threat in Enterobacteriaceae, pseudomonas and acinetobacter. Trends Microbiol.

[5] Zimlichman E, Henderson D, Tamir O, Franz C, Song P, Yamin CK (2013). Health care-associated infections: a meta-analysis of costs and financial impact on the US health care system. JAMA Intern Med.

[6] Nathwani D, Raman G, Sulham K, Gavaghan M, Menon V (2014). Clinical and economic consequences of hospital-acquired resistant and multidrug-resistant Pseudomonas aeruginosa infections: a systematic review and meta-analysis. Antimicrob Resist Infect Control.

[7] Moher D, Liberati A, Tetzlaff J, Altman DG (2010). Preferred reporting items for systematic reviews and meta-analyses: the PRISMA statement. BMJ.

[8] DerSimonian R, Laird N (1986). Meta-analysis in clinical trials. Control Clin Trials.

[9] Huedo-Medina TB, Sanchez-Meca J, Marin-Martinez F, Botella J (2006). Assessing heterogeneity in meta-analysis: Q statistic or I2 index?. Psychol Methods.

[10] Egger M, Davey SG, Schneider M, Minder C (1997). Bias in meta-analysis detected by a simple, graphical test. BMJ.

[11] Wells G, Shea B, O’Connell D, Peterson J, Welch V, Losos M, Shea B, O’Connell D, Peterson J, Welch V, Losos M, Tugwell P: The Newcastle-Ottawa Scale (NOS) for assessing the quality of nonrandomised studies in meta-analyses. http://www.ohri.ca/programs/clinical_epidemiology/oxford.asp. Published 2007. Accessed January 12, 2015

[12] Cordery RJ, Roberts CH, Cooper SJ, Bellinghan G, Shetty N (2008). Evaluation of risk factors for the acquisition of bloodstream infections with extended-spectrum beta-lactamase-producing Escherichia coli and Klebsiella species in the intensive care unit; antibiotic management and clinical outcome. J Hosp Infect.

[13] Du B, Long Y, Liu H, Chen D, Liu D, Xu Y (2002). Extended-spectrum beta-lactamase-producing Escherichia coli and Klebsiella pneumoniae bloodstream infection: risk factors and clinical outcome. Intensive Care Med.

[14] de Gouvea EF, Martins IS, Halpern M, Ferreira AL, Basto ST, Goncalves RT (2012). The influence of carbapenem resistance on mortality in solid organ transplant recipients with Acinetobacter baumannii infection. BMC Infect Dis.

[15] Edis EC, Hatipoglu ON, Tansel O, Sut N (2010). Acinetobacter pneumonia: Is the outcome different from the pneumonias caused by other agents. Ann Thoracic Med.

[16] Erbay A, Idil A, Gozel MG, Mumcuoglu I, Balaban N (2009). Impact of early appropriate antimicrobial therapy on survival in Acinetobacter baumannii bloodstream infections. Int J Antimicrob Agents.

[17] Falagas ME, Kasiakou SK, Rafailidis PI, Zouglakis G, Morfou P (2006). Comparison of mortality of patients with Acinetobacter baumannii bacteraemia receiving appropriate and inappropriate empirical therapy. J Antimicrob Chemother.

[18] Garnacho-Montero J, Sa-Borges M, Sole-Violan J, Barcenilla F, Escoresca-Ortega A, Ochoa M (2007). Optimal management therapy for Pseudomonas aeruginosa ventilator-associated pneumonia: an observational, multicenter study comparing monotherapy with combination antibiotic therapy. Crit Care Med.

[19] Gozel M, Erbay A, Bodur H, Eren S, Balaban N (2012). Risk factors for mortality in patients with nosocomial gram-negative bacteremia. Turkiye Klinikleri J Med Sci.

[20] Huang ST, Chiang MC, Kuo SC, Lee YT, Chiang TH, Yang SP (2012). Risk factors and clinical outcomes of patients with carbapenem-resistant Acinetobacter baumannii bacteremia. J Microbiol Immunol Infect.

[21] Jamulitrat S, Arunpan P, Phainuphong P (2009). Attributable mortality of imipenem-resistant nosocomial Acinetobacter baumannii bloodstream infection. J Med Assoc Thai.

[22] Joung MK, Kwon KT, Kang CI, Cheong HS, Rhee JY, Jung DS (2010). Impact of inappropriate antimicrobial therapy on outcome in patients with hospital-acquired pneumonia caused by Acinetobacter baumannii. J Infect.

[23] Kang CI, Kim SH, Park WB, Lee KD, Kim HB, Kim EC (2005). Bloodstream infections caused by antibiotic-resistant gram-negative bacilli: risk factors for mortality and impact of inappropriate initial antimicrobial therapy on outcome. Antimicrob Agents Chemother.

[24] Kim YJ, Kim SI, Hong KW, Kim YR, Park YJ, Kang MW (2012). Risk factors for mortality in patients with carbapenem-resistant Acinetobacter baumannii bacteremia: impact of appropriate antimicrobial therapy. J Korean Med Sci.

[25] Kollef KE, Schramm GE, Wills AR, Reichley RM, Micek ST, Kollef MH (2008). Predictors of 30-day mortality and hospital costs in patients with ventilator-associated pneumonia attributed to potentially antibiotic-resistant gram-negative bacteria. Chest.

[26] Kuo SC, Lee YT, Yang SP, Chiang MC, Lin YT, Tseng FC (2013). Evaluation of the effect of appropriate antimicrobial therapy on mortality associated with Acinetobacter nosocomialis bacteraemia. Clin Microbiol Infect.

[27] Lee HY, Chen CL, Wu SR, Huang CW, Chiu CH (2014). Risk factors and outcome analysis of acinetobacter baumannii complex bacteremia in critical patients. Crit Care Med.

[28] Lin YT, Chiu CH, Chan YJ, Lin ML, Yu KW, Wang FD (2009). Clinical and microbiological analysis of Elizabethkingia meningoseptica bacteremia in adult patients in Taiwan. Scand J Infect Dis.

[29] Lin YT, Liu CJ, Fung CP, Tzeng CH (2011). Nosocomial Klebsiella pneumoniae bacteraemia in adult cancer patients--characteristics of neutropenic and non-neutropenic patients. Scand J Infect Dis.

[30] Lodise TP, Patel N, Kwa A, Graves J, Furuno JP, Graffunder E (2007). Predictors of 30-day mortality among patients with Pseudomonas aeruginosa bloodstream infections: impact of delayed appropriate antibiotic selection. Antimicrob Agents Chemother.

[31] Lye DC, Earnest A, Ling ML, Lee TE, Yong HC, Fisher DA (2012). The impact of multidrug resistance in healthcare-associated and nosocomial Gram-negative bacteraemia on mortality and length of stay: cohort study. Clin Microbiol Infect.

[32] Mehta A, Kumar V, Kumari I, Nair S, Dinesh K, Singh S (2012). Risk factors for mortality in Acinetobacter calcoaceticus-baumannii bacteraemia. Asian Pac J Trop Biomed.

[33] Metan G, Zarakolu P, Cakir B, Hascelik G, Uzun O (2005). Clinical outcomes and therapeutic options of bloodstream infections caused by extended-spectrum beta-lactamase-producing Escherichia coli. Int J Antimicrob Agents.

[34] Metan G, Sariguzel F, Sumerkan B (2009). Factors influencing survival in patients with multi-drug-resistant Acinetobacter bacteraemia. Eur J Intern Med.

[35] Metan G, Demiraslan H, Kaynar LG, Zararsiz G, Alp E, Eser B (2013). Factors influencing the early mortality in haematological malignancy patients with nosocomial Gram negative bacilli bacteraemia: a retrospective analysis of 154 cases. Braz J Infect Dis.

[36] Micek ST, Lloyd AE, Ritchie DJ, Reichley RM, Fraser VJ, Kollef MH (2005). Pseudomonas aeruginosa bloodstream infection: importance of appropriate initial antimicrobial treatment. Antimicrob Agents Chemother.

[37] Navarro-San FC, Mora-Rillo M, Romero-Gomez MP, Moreno-Ramos F, Rico-Nieto A, Ruiz-Carrascoso G (2013). Bacteraemia due to OXA-48-carbapenemase-producing Enterobacteriaceae: a major clinical challenge. Clin Microbiol Infect.

[38] Ng E, Earnest A, Lye DC, Ling ML, Ding Y, Hsu LY (2012). The excess financial burden of multidrug resistance in severe gram-negative infections in Singaporean hospitals. Ann Acad Med Singap.

[39] Park KH, Shin JH, Lee SY, Kim SH, Jang MO, Kang SJ (2013). The clinical characteristics, carbapenem resistance, and outcome of Acinetobacter bacteremia according to genospecies. PLoS ONE [Electronic Resource].

[40] Pena C, Gudiol C, Calatayud L, Tubau F, Dominguez MA, Pujol M (2008). Infections due to Escherichia coli producing extended-spectrum beta-lactamase among hospitalised patients: factors influencing mortality. J Hosp Infect.

[41] Pena C, Gomez-Zorrilla S, Oriol I, Tubau F, Dominguez MA, Pujol M (2013). Impact of multidrug resistance on Pseudomonas aeruginosa ventilator-associated pneumonia outcome: predictors of early and crude mortality. Eur J Clin Microbiol.

[42] Rodriguez-Bano J, Picon E, Gijon P, Hernandez JR, Cisneros JM, Pena C (2010). Risk factors and prognosis of nosocomial bloodstream infections caused by extended-spectrum-beta-lactamase-producing Escherichia coli. J Clin Microbiol.

[43] Su TY, Ye JJ, Hsu PC, Wu HF, Chia JH, Lee MH (2015). Clinical characteristics and risk factors for mortality in cefepime-resistant Pseudomonas aeruginosa bacteremia. J Microbiol Immunol Infect.

[44] Tam VH, Rogers CA, Chang KT, Weston JS, Caeiro JP, Garey KW (2010). Impact of multidrug-resistant Pseudomonas aeruginosa bacteremia on patient outcomes. Antimicrob Agents Chemother.

[45] Thom KA, Shardell MD, Osih RB, Schweizer ML, Furuno JP, Perencevich EN (2008). Controlling for severity of illness in outcome studies involving infectious diseases: impact of measurement at different time points. Infect Control Hosp Epidemiol.

[46] Thom KA, Schweizer ML, Osih RB, McGregor JC, Furuno JP, Perencevich EN (2008). Impact of empiric antimicrobial therapy on outcomes in patients with Escherichia coli and Klebsiella pneumoniae bacteremia: a cohort study. BMC Infect Dis.

[47] Tumbarello M, Viale P, Viscoli C, Trecarichi EM, Tumietto F, Marchese A (2012). Predictors of mortality in bloodstream infections caused by Klebsiella pneumoniae carbapenemase-producing K. pneumoniae: importance of combination therapy. Clin Infect Dis.

[48] Tumbarello M, De PG, Trecarichi EM, Spanu T, Antonicelli F, Maviglia R (2013). Clinical outcomes of Pseudomonas aeruginosa pneumonia in intensive care unit patients. Intensive Care Med.

[49] Tuon FF, Kruger M, Terreri M, Penteado-Filho SR, Gortz L (2011). Klebsiella ESBL bacteremia-mortality and risk factors. Braz J Infect Dis.

[50] Tuon FF, Gortz LW, Rocha JL (2012). Risk factors for pan-resistant Pseudomonas aeruginosa bacteremia and the adequacy of antibiotic therapy. Braz J Infect Dis.

[51] Vitkauskiene A, Skrodeniene E, Dambrauskiene A, Macas A, Sakalauskas R (2010). Pseudomonas aeruginosa bacteremia: resistance to antibiotics, risk factors, and patient mortality. Medicina (Kaunas).

[52] Yang YS, Lee YT, Tsai WC, Kuo SC, Sun JR, Yang CH (2013). Comparison between bacteremia caused by carbapenem resistant Acinetobacter baumannii and Acinetobacter nosocomialis. BMC Infect Dis.

[53] Zarkotou O, Pournaras S, Tselioti P, Dragoumanos V, Pitiriga V, Ranellou K (2011). Predictors of mortality in patients with bloodstream infections caused by KPC-producing Klebsiella pneumoniae and impact of appropriate antimicrobial treatment. Clin Microbiol Infect.

[54] Apisarnthanarak A, Kiratisin P, Mundy LM (2008). Predictors of mortality from community-onset bloodstream infections due to extended-spectrum beta-lactamase-producing Escherichia coli and Klebsiella pneumoniae. Infect Control Hosp Epidemiol.

[55] Bailey AM, Weant KA, Baker SN (2013). Prevalence and risk factor analysis of resistant Escherichia coli urinary tract infections in the emergency department. Pharm Pract.

[56] Bare M, Castells X, Garcia A, Riu M, Comas M, Egea MJ (2006). Importance of appropriateness of empiric antibiotic therapy on clinical outcomes in intra-abdominal infections. Int J Technol Assess Health Care.

[57] Lautenbach E, Patel JB, Bilker WB, Edelstein PH, Fishman NO (2001). Extended-spectrum beta-lactamase-producing Escherichia coli and Klebsiella pneumoniae: risk factors for infection and impact of resistance on outcomes. Clin Infect Dis.

[58] Lee NY, Lee HC, Ko NY, Chang CM, Shih HI, Wu CJ (2007). Clinical and economic impact of multidrug resistance in nosocomial Acinetobacter baumannii bacteremia. Infect Control Hosp Epidemiol.

[59] Lee SS, Kim Y, Chung DR (2011). Impact of discordant empirical therapy on outcome of community-acquired bacteremic acute pyelonephritis. J Infect.

[60] MacVane SH, Tuttle LO, Nicolau DP (2014). Impact of extended-spectrum beta-lactamase-producing organisms on clinical and economic outcomes in patients with urinary tract infection. J Hosp Med (Online).

[61] Osih RB, McGregor JC, Rich SE, Moore AC, Furuno JP, Perencevich EN (2007). Impact of empiric antibiotic therapy on outcomes in patients with Pseudomonas aeruginosa bacteremia. Antimicrob Agents Chemother.

[62] Schwaber MJ, Navon-Venezia S, Kaye KS, Ben-Ami R, Schwartz D, Carmeli Y (2006). Clinical and economic impact of bacteremia with extended- spectrum-beta-lactamase-producing Enterobacteriaceae. Antimicrob Agents Chemother.

[63] Shorr AF, Micek ST, Welch EC, Doherty JA, Reichley RM, Kollef MH (2011). Inappropriate antibiotic therapy in Gram-negative sepsis increases hospital length of stay. Crit Care Med.

[64] Sturkenboom MC, Goettsch WG, Picelli G, in ‘t Veld B, Yin DD, De Jong RB (2005). Inappropriate initial treatment of secondary intra-abdominal infections leads to increased risk of clinical failure and costs. Br J Clin Pharmacol.

[65] Tumbarello M, Spanu T, Di BR, Marchetti M, Ruggeri M, Trecarichi EM (2010). Costs of bloodstream infections caused by Escherichia coli and influence of extended-spectrum-beta-lactamase production and inadequate initial antibiotic therapy. Antimicrob Agents Chemother.

[66] Yang YS, Ku CH, Lin JC, Shang ST, Chiu CH, Yeh KM (2010). Impact of Extended-spectrum beta-lactamase-producing Escherichia coli and Klebsiella pneumoniae on the outcome of community-onset bacteremic urinary tract infections. J Microbiol Immunol Infect.

[67] Chuang YC, Chang SC, Wang WK (2012). Using the rate of bacterial clearance determined by real-time polymerase chain reaction as a timely surrogate marker to evaluate the appropriateness of antibiotic usage in critical patients with Acinetobacter baumannii bacteremia. [Erratum appears in Crit Care Med.

[68] O’Neal CS, O’Neal HR, Daniels TL, Talbot TR (2012). Treatment outcomes in patients with third-generation cephalosporin-resistant Enterobacter bacteremia. Scandinavian J Infect Dis.

[69] Santimaleeworagun W, Wongpoowarak P, Chayakul P, Pattharachayakul S, Tansakul P, Garey KW (2011). Clinical outcomes of patients infected with carbapenem-resistant Acinetobacter baumannii treated with single or combination antibiotic therapy. J Med Assoc Thai.

[70] Shime N, Kosaka T, Fujita N (2013). De-escalation of antimicrobial therapy for bacteraemia due to difficult-to-treat Gram-negative bacilli. Infection.

[71] Davies J, Davies D (2010). Origins and evolution of antibiotic resistance. Microbiol Mol Biol Rev.

[72] Threat Report 2013: Antibiotic/Antimicrobial Resistance. Centers for Disease Control and Prevention. Available online: http://www.cdc.gov/drugresistance/threat-report-2013/. Published 2013. Accessed on January 12, 2015

[73] Pallett A, Hand K (2010). Complicated urinary tract infections: practical solutions for the treatment of multiresistant Gram-negative bacteria. J Antimicrob Chemother.

[74] Bader MS, Hawboldt J, Brooks A (2010). Management of complicated urinary tract infections in the era of antimicrobial resistance. Postgrad Med.

[75] Zilberberg MD, Shorr AF, Micek ST, Vazquez-Guillamet C, Kollef MH (2014). Multi-drug resistance, inappropriate initial antibiotic therapy and mortality in Gram-negative severe sepsis and septic shock: a retrospective cohort study. Crit Care.

[76] The bacterial challenge: time to react. Available online: http://ecdc.europa.eu/en/publications/Publications/0909_TER_The_Bacterial_Challenge_Time_to_React.pdf. Published on 2009. Accessed on January 12, 2015

[77] Lautenbach E, Perencevich EN (2014). Addressing the emergence and impact of multidrug-resistant gram-negative organisms: a critical focus for the next decade. Infect Control Hosp Epidemiol.

