# Supplementary material for: Appropriate initial antibiotic therapy in hospitalized patients with gram-negative infections: systematic review and meta-analysis
Source: BMC Infect Dis. 2015 Sep 30;15:395. doi: 10.1186/s12879-015-1123-5 (PMC4589179; doi:10.1186/s12879-015-1123-5)
Supplement: Additional file 1: Table S1. — Characteristics of included studies reporting mortality outcomes. (DOCX 98 kb) [file 12879_2015_1123_MOESM1_ESM.docx]

Additional File 1.

**Table 1 - Characteristics of included studies reporting mortality outcomes**

| **Author Year (Country)** | **Infecting pathogen** | **Baseline Comorbidity Mean Score** | **Appropriate IAT definition** | **Adjusted covariates for Mortality outcome** | **Total N** | **Mortality N (%)** |
| --- | --- | --- | --- | --- | --- | --- |
| Cordery 2008 (UK)[12] | ESBL E.coli; Klebsiella spp | 65% had APACHE ≥20 or SOFA ≥5 | Antibiotic administration ≤24 h after blood cultures | Disease severity, ESBL infection | 55 | 25 (45) |
| Du 2002 (China)[13] | E.coli; K.pneumoniae | APACHE II 15.6 | Subsequent identification of the infecting pathogen’s in vitro susceptibility to the IV antibiotic (timeframe NR) | None | 85 | 21 (25) |
| Edis 2010 (Turkey)[15] | Acinetobacter spp | NR | Antibiotic administration ≤72 h appropriate to the antibiogram of the identified pathogen | None | 63 | 41 (65) |
| Erbay 2009 (Turkey)[16] | A.baumannii | APACHE II score 13.3 | Antibiotic administration using proper dosage and route ≤48 h after a blood culture, with at least one active antibiotic susceptible in vitro | Age>65 years, septic shock, mechanical ventilation | 103 | 56 (54) |
| Falagas 2006 (Greece)[17] | A.baumannii | APACHE II score 16.2 | Antibiotic administration ≤72 h after a blood culture to which the infecting pathogen was susceptible in vitro | None | 40 | 19 (48) |
| Ferraz de Gouvea 2012 (Brazil)[14] | A.baumannii | NR | Antibiotic administration ≤48 h after diagnosis | ICU Infection, mechanical ventilation, resistance to carbapenem | 49 | 29 (59) |
| Garnacho-Montero 2007 (Spain)[18] | P.aeruginosa | APACHE II 19.1 | Administered at least one effective antibiotic based on in vitro susceptibility (timeframe NR) | Age; chronic cardiac insufficiency | 183 | 77 (42) |
| Gozel 2012 (Turkey)[19] | Ecoli; Klebsiella spp.; P.aeruginosa; Acinetobacter spp.; Enterobacter spp.; S. maltophilia | APACHE II score survivors 17.2; Non-survivors 21.4 | Administered antibiotic to which the infecting pathogens were susceptible (timeframe NR) | APACHE II score>20; Total parenteral nutrition; Unconsciousness, Thrombocytopenia | 240 | 92 (38) |
| Huang 2012 (Taiwan)[20] | A.baumannii | APACHE II score >20 | Antibiotic administration ≤72 h of blood collection to which the infecting pathogen was susceptible | APACHE II score>20; shock | 226 | 56 (25) |
| Jamulitrat 2010 (Thailand)[21] | A.baumannii | SOFA: 5 | Administered antibiotic based on in vitro susceptibility ≤72 h of blood collection | None | 198 | 61 (31) |
| Joung 2010 (South Korea)[22] | A.baumannii | APACHE II 22.3 Charlson 2.5 | Administered antibiotic ≤24 h that included at least one active antibiotic according to the sensitivity test | APACHE II score>=20; MDR; PDR | 116 | 44 (38) |
| Kang 2005 (South Korea)[23] | E.coli; K.pneumonia; Enterobacter spp; P.aeruginosa | APACHE II 11.2 | Administered antibiotic ≤24 h of culture samples via an appropriate route and dosage to which the infecting pathogens were susceptible in vitro | Septic shock; bacteremia; P.aeruginosa infection; increasing APACHE score | 286 | 95 (33) |
| Kim 2012 (South Korea)[24] | Carbapenem-resistant Acinetobacter. baumannii | APACHE II 12.4 Charlson 2.7 | Antibiotic administration ≤24 h of blood culture via an appropriate route and dosage based on in vitro susceptibility | septic shock; carbapenem-resistance; pneumonia | 95 | 30 (32) |
| Kollef 2008 (USA)[25] | Antibiotic-resistant Gram-negative bacteria | APACHE II Non-survivors 23.5; Survivors 15.5 | NR | APACHE II score; Septic shock | 76 | 19 (25) |
| Kuo 2012 (Taiwan)[26] | A.nosocomialis | APACHE II Non-survivors 31; Survivors 18 | Antibiotic IV administration using proper dosage ≤48 h after a blood culture with ≥ one active antibiotic that is in vitro susceptible | None | 266 | 25 (9) |
| Lee 2014 (Taiwan)[27] | A. baumannii | Charlson score: 3  APACHE II: Non-survivors 24.5; Survivors 18 | Administration ≤48 hr of onset with at least one antimicrobial agent, except aminoglycoside, susceptible in vitro | imipenem-resistant infection, higher Pitt bacteremia score, and catheter-related infection or urinary tract infection | 298 | 100 (34) |
| Lin 2009 (Taiwan)[28] | C.meningosepticum | NR | Intravenous administration with appropriate dose ≤ 72 h of diagnosis of antibiotic to which the microorganism was susceptible | Septic shock | 32 | 13 (41) |
| Lin 2011 (Taiwan)[29] | K.pneumonia | NR | Administration of in vitro active antibiotic after the index blood culture and before availability of susceptibility results (timeframe NR) | None | 189 | 48 (25) |
| Lodise 2007 (USA)[30] | P.aeruginosa | APACHE II 17.1 | Administration of at least one intravenous antibiotic to which the pathogen was susceptible (timeframe for analysis >52h) | APACHE-II score; ICU at onset; mechanical ventilation at onset; decubitus ulcers | 100 | 31 (31) |
| Lye 2012 (Singapore)[31] | E.coli; K.pneumonia; P.aeruginosa; A.baumannii; Enterobacter spp; Proteus | APACHE II* Nonsurvivors 14,survivors 9; Charlson* Nonsurvivors 9, survivors 7 | Prescribed appropriate doses ≤24 h that had in vitro activity against all isolated organisms based on Sanford Guide to Antimicrobial Therapy of antibiotics | Male gender; surgical discipline; higher charlson comorbidity index; Higher APACHE II score; Pneumonia or UTI; ICU stay | 671 | 146 (22) |
| Mehta 2012 (India)[32] | Acinetobacter spp | NR | Administration of at least one antibiotic within 24-48 h that was sensitive in vitro | Age, diabetes, end stage renal disease, ventilator support, platelet count<1.5 lac, S. creatinine 1.5, Prothrombin time>15 sec, Blood urea >40mg/dL, Carbapenem resistance | 81 | 36 (44) |
| Metan 2005 (Turkey)[33] | ESBL E.coli | NR | Antibiotics active in vitro (except ceftriaxone, cefotaxime and ceftazidime) administered with appropriate dosage and route (timeframe NR) | None | 53 | 14 (26) |
| Metan 2009 (Turkey)[34] | Acinetobacter spp | NR | Administration ≤24 h of blood culture via appropriate dosage and route of one or more agents active against infecting pathogen | None | 100 | 63 (63) |
| Metan 2013 (Turkey)[35] | E.coli Klebsielle spp.; P.aeruginosa; Acinetobacter spp; S. maltophilia | NR | Administration with proper dosage and route of administration ≤24 h after blood sample collection that was active in vitro (except ceftriaxone, cefotaxime and ceftazidime) | None | 154 | 30 (20) |
| Micek 2005 (USA)[36] | P.aeruginosa | SAP Survivors 10.9 Nonsurvivors 13.8 | A positive blood culture result that was effectively treated with antibiotic at the time the pathogen and its susceptibility were known (timeframe NR) | Race; infection source; acute renal failure; patient location; respiratory failure; circulatory shock; and SAP score | 305 | 64 (21) |
| Navarro-San Francisco 2012 (Spain)[37] | OXA-48-carbapenemase-producing K.pneumonia; E.coli | Charlson* 5 | Administration of at least one active agent against the isolate that is susceptible (timeframe NR) | None | 40 | 20 (50) |
| Park 2013 (South Korea)[39] | Acinetobacter spp | APACHE II: 18 | Administration ≤48 h of at least one antibiotic to which the pathogen was susceptible (timeframe NR) | None | 180 | 49 (27) |
| Pena 2008 (Spain)[40] | ESBL and non-ESBL E.coli | NR | Administration ≤48 h of at least one antibiotic active in vitro against the infecting microorganism | Source of infection; type of ESBL pathogen | 191 | 22 (12) |
| Pena 2013 (Spain)[41] | P.aeruginosa | Charlson: Non-survivor 2.6 Survivor 2.2 | Administration ≤24 h of antimicrobial therapy to which P. aeruginosa isolate was susceptible. | None | 91 | 47 (52) |
| Rodriguez-Bano 2010 (Spain)[42] | ESBL E.coli | Charlson>2: 42% | Administration ≤24 h after blood sample for culture was draw of active antibiotic at the recommended dosages | None | 96 | 29 (30) |
| Su 2013 (Taiwan)[43] | P.aeruginosa | Pittsburg 5.0 | Antibiotic agents with correct dosage used ≤72 h and proved to be effective in vitro against the infecting pathogen. | Pittsburgh bacteremia score | 78 | 51 (65) |
| Tam 2010[44] | P.aeruginosa | APACHE II: MDR 14.9; MDS 12.2 | Administration with appropriate dosage ≤24 h of sample culture was obtained to which the isolate was found to be susceptible on the final susceptibility report | Multidrug resistance; APACHE II score; renal condition; immunosuppression; source of bacteremia | 109 | 28 (26) |
| Thom 2008 (USA)[45] | E.coli; Klebsiella spp.; P.aeruginosa | Acute physiology score 19.2 | Administration between 8-24 h with antimicrobials to which the specific isolate displayed in vitro susceptibility | Modified APS at first time point; change in severity-of-illness scores | 328 | 96 (29) |
| Tumbarello 2012 (Italy)[47] | KPC producing K.pneumoniae | Charlson* Nonsurvivors 2; survivors 2 APACHE Nonsurvivors 40; survivors 24 | Administration of at least one drug displaying in vitro activity against the infecting pathogen (timeframe NR) | septic shock at BSI onset, high APACHE III scores | 125 | 52 (42) |
| Tumbarello 2013 (Italy)[48] | P.aeruginosa | SOFA at pneumonia onset: 7; SAPS II on admission: 43  43 | Administration of at least one agent displaying in vitro activity against the isolated pathogen (timeframe NR) | Diabetes, higher SAPS II, and older age | 99 | 39 (39) |
| Tuon 2011 (Brazil)[49] | ESBL K. pneumoniae | NR | Administration ≤48 h of diagnosis of antibiotic to which the isolated pathogen was susceptible | None | 104 | 48 (46) |
| Tuon 2012 (Brazil)[50] | P.aeruginoas | NR | Infecting pathogen was susceptible to the antibiotic used/started in ≤24 h after blood collection | None | 77 | 38 (49) |
| Vitkauskiene 2010 (Lithuania)[51] | P.aeruginoas | NR | Administration of antibiotic to which the pathogen was found to be susceptible (timeframe NR) | None | 80 | 47 (59) |
| Yang 2013 (Taiwan)[52] | Acinetobacter spp. | APACHE II: 26 | Antibiotic administration with an approved route and dosage, ≥48 h after the onset of bacteremia, to which the causative pathogen was susceptible | None | 135 | 64 (47) |
| Zarkotou 2011 (Greece)[53] | KPC producing K.pneumonia | APACHE II 21 | Administration ≤24 h of infection onset of in vitro active antimicrobials against the study isolates | None | 53 | 18 (34) |

Abbreviations: A.baumannii, Acinetobacter baumannii; APACHE, acute physiology and chronic health evaluations; ESBL, Extended spectrum beta-lactamase; ICU, Intensive care unit; IAT, Inappropriate initial antibiotic therapy; IRAB, Imipenem-Resistant Acinetobacter Baumannii; KPC, Klebsiella pneumoniae carbapenemases; MDR, Multi-drug Resistant; MDS, Multi-drug susceptible; NR, Not Reported; SD, Standard deviation; S.maltophilia, Stenotrophomonas maltophilia; SAP, simplified acute physiology, SOFA, Sequential Organ Failure Assessment score
