# Supplementary material for: Appropriate initial antibiotic therapy in hospitalized patients with gram-negative infections: systematic review and meta-analysis
Source: BMC Infect Dis. 2015 Sep 30;15:395. doi: 10.1186/s12879-015-1123-5 (PMC4589179; doi:10.1186/s12879-015-1123-5)
Supplement: Additional file 2: Table S2. — Additional characteristics of included studies reporting mortality outcomes. (DOCX 95 kb) [file 12879_2015_1123_MOESM2_ESM.docx]

Additional File 2.

**Table 2 – Additional characteristics of included studies reporting mortality outcomes**

| **Author Year** | **Country of Study** | **Study Design** | **Hospital Setting** | **Enrollment Period** | **Follow-up** | **Total N** | **Mean Age (SD), yrs** | **Median Age (Age Range)** | **Male %** |
| --- | --- | --- | --- | --- | --- | --- | --- | --- | --- |
| Cordery 2008[12] | UK | Retrospective | Academic | 2004–2006 | NR | 16 | NR | <40: 12.5%; ≥40 87.5% | 75 |
| Du 2002[13] | China | Retrospective | Academic | 1997-1999 | NR | 85 | 48.7 (19.7) | NR | 47 |
| Edis 2010[15] | Turkey | Prospective | Academic | 2005–2006 | 6 weeks | 63 | 64.4 (13.9) | NR | 60.3 |
| Erbay 2009[16] | Turkey | Retrospective | Tertiary care | 2005–2008 | NR | 103 | Age >65: 34% | NR | 61.2 |
| Falagas 2006[17] | Greece | Retrospective | Tertiary-care | 2002–2004 | NR | 40 | Inapprop 68.5 (15.3) Approp 57.4 (18.8) | NR | 50 |
| Ferraz de Gouvea 2012[14] | Brazil | Retrospective | Academic | 2002–2009 | Until death or hospital discharge | 49 | NR | Carbapenem-resistant 48 (28–63); Carbapenem-susceptible 48 (37–58) | 59.2 |
| Garnacho-Montero 2007[18] | Spain | Retrospective | Academic ICUs | 2002–2006 | Until death or hospital discharge | 183 | 56.3 (17.6) | NR | 74.9 |
| Gozel 2012[19] | Turkey | Prospective | Tertiary care | 2006–2008 | NR | 253 | 54.5 (20) | NR | 63 |
| Huang 2012[20] | Taiwan | Retrospective | Veterans General hospital, ICU | 2002–2007 | 14 days | 226 (CRAB=62; CSAB=164) | CRAB=69.6 (15.8) CSAB=68.5 (17.6) | NR | 77 |
| Jamulitrat 2010[21] | Thailand | Restrospective | Academic | 2004–2007 | NR | 198 (67 IRAB and 131 ISAB) | IRAB: 56.1 (19.4)  ISAB: 50.8 (19.7) | NR | IRAB: 44.8  ISAB: 59.5 |
| Joung 2010[22] | South Korea | Retrospective | Academic | 2000–2006 | NR | 116 | 60.4 (16.3) | 16–94 | 77.6 |
| Kang 2005[23] | Korea | Retrospective | Academic | 1998–2002 | NR | 286 | 55 (16) | (16–95) | 64 |
| Kim 2012[24] | South Korea | Retrospective | Tertiary care | 2007–2010 | NR | 95 | 58.4 (20.9) | NR | 53.7 |
| Kollef 2008[25] | USA | Retrospective | Tertiary care | 2002–2006 | NR | 76 | 56.6 (16.9) | NR | 65.8 |
| Kuo 2012[26] | Taiwan | Retrospective | Academic | 2000–2008 | NR | 266 | 72.6 | NR | 73.8 |
| Lee 2014[27] | Taiwan | Retrospective | Tertiary care | 2009–2010 | NR | 298 | NR | Non-survivors 67(57–78); Survivors 63 (53–74) | 57.4 |
| Lin 2009[28] | Taiwan | Retrospective | Academic | 2004–2007 | NR | 32 | 72 (NR) | 78.5 (22–89) | 81 |
| Lin 2011[29] | Taiwan | Retrospective | Tertiary care | 2007–2009 | NR | 189 | Age >65 y 54% | NR | 70 |
| Lodise 2007[30] | USA | Retrospective | Academic | 2001–2006 | NR | 100 | 57.8 (17.9) | NR | 55 |
| Lye 2012[31] | Singapore | Retrospective | Tertiary care | 2007–2009 | Until discharge or death | 675 | NR | Survivors 67 (54–77) Nonsurvivors 71 (57–79) | 52 |
| Mehta 2012[32] | India | Retrospective | Tertiary care | 2008–2009 | NR | 81 | 52.6 (NR) | NR (1–95) | 71.6 |
| Metan 2005[33] | Turkey | Retrospective | NR | 2003–2005 | NR | 53 | 54.6 (17.5) | NR | 54.7 |
| Metan 2009[34] | Turkey | Prospective | Tertiary care | 2007–2008 | NR | 100 | NR | 58 (17–83) | 54 |
| Metan 2013[35] | Turkey | Retrospective | Academic | 2006–2011 | NR | 154 | NR | 40.7 (16–76) | 55.8 |
| Micek 2005[36] | USA | Retrospective | Academic | 1997–2002 | NR | 305 | 58.2 (17.8) | (16–97) | 56.7 |
| Navarro-San Francisco 2012[37] | Spain | Prospective | Academic | 2010–2012 | None | 40 | 70 | 73 (38–92) | 57.5 |
| Park 2013[39] | Republic of Korea | Retrospective | Academic | 2003–2010 | 30 days | 180 | *Acinetobacter baumannii* (n=90) 60 (17); Non-*A. baumanni* ACB complex (n=90) = 58 (18) | NR | 64 |
| Pena 2008[40] | Spain | Retrospective matched cohort | Academic | 1996–2003 | 30 days | 200 | 61.1 (15.5) | NR | 54 |
| Pena 2013[41] | Spain | Retrospective | Academic | 2006–2011 | NR | 83 | 69.39 (14.15) | NR | 78 |
| Rodriguez-Bano 2010[42] | Spain | Retrospective | Multicenter Tertiary care | 2004–2006 | NR | 96 | NR  age >65, 47% | NR | 56 |
| Su 2013[43] | Taiwan | Retrospective | Academic | 2006–2011 | NR | 78 | 72.2 (14.1) | NR | 59 |
| Tam 2010[44] | USA | Retrospective | Tertiary care | 2005–2008 | NR | 109 | MDR 57.4 (10.7)  MDS 61.6 (14.8) | NR | 49 |
| Thom 2008[45] | USA | Retrospective | Academic | 2001–2005 | NR | 328 | 57 (16) | NR | 62 |
| Tumbarello 2012[47] | Italy | Retrospective | Multicenter Academic | 2010–2011 | NR | 125 | Survivors 62.9 (16.5)  Nonsurvivors 61.5 (14.3) | NR | 58 |
| Tumbarello 2013[48] | Italy | Retrospective | Academic ICUs | 2008–2010 | NR | 110 | NR | IIAT group median 70 (52–79)  AIAT median 64 (40–75) | 65 |
| Tuon 2011[49] | Brazil | Retrospective | Academic | 2006–2009 | 30-day mortality | 104 | ESBL: 38.3 (21.7) non-ESBL: 44.2 (25.3) | 43 (12–86) | 57 |
| Tuon 2012[50] | Brazil | Retrospective | Tertiary care | 2006–2009 | 30 days | 77 | Carbapenem-resistant 46.4 +/- 22.71 [n=29]  Carbapenem-susceptible 49.0 (20.4) [n=48] | NR | 73 |
| Vitkauskiene 2010[51] | Lithuania | Retrospective | Academic | 2003–2007 | NR | 80 | 56.7 (16.7) | NR | 70 |
| Yang 2013[52] | Taiwan | Retrospective | Academic | 2000–2008 | NR | 135 | NR | 75 (61–82); 76 (62–81) | 74.1 |
| Zarkotou 2011[53] | Greece | Retrospective | Tertiary care | 2008–2010 | Until discharge or death | 53 | 63.8 (19.9) | NR | 62.3 |

Abbreviations: ACB, Acinetobacter calcoaceticus–baumannii; AIAT, Appropriate initial antibiotic therapy; CRAB, Carbapenem-resistant Acinetobacter baumannii; CSAB, Carbapenem-susceptible Acinetobacter baumannii; ESBL, Extended spectrum beta-lactamase; ICU, Intensive care unit; IIAT, Inappropriate initial antibiotic therapy; IRAB, Imipenem-Resistant Acinetobacter Baumannii; MDR, Multi-drug Resistant; MDS, Multi-drug susceptible; NR, Not Reported; SD, Standard deviation
