# Supplementary material for: Appropriate initial antibiotic therapy in hospitalized patients with gram-negative infections: systematic review and meta-analysis
Source: BMC Infect Dis. 2015 Sep 30;15:395. doi: 10.1186/s12879-015-1123-5 (PMC4589179; doi:10.1186/s12879-015-1123-5)
Supplement: Additional file 3: Table S3. — Study characteristics and results of economic outcomes. Table S4. Study characteristics and results of length of stay (DOCX 74 kb) [file 12879_2015_1123_MOESM3_ESM.docx]

Additional File 3.

**Table 1 - Study characteristics and results of economic outcomes**

| **Author Year** | **Country; Recruited Yr** | **Infection**  **site** | **Pathogen sp.** | **Total N** | **Receiving IAT** | **Cost Definition** | **Result**  **IAT** | **Result**  **AAT** | **P value** |
| --- | --- | --- | --- | --- | --- | --- | --- | --- | --- |
| **Direct Evidence** |  |  |  |  |  |  |  |  |  |
| Ng 2012[38]  (same study as Lye 2012) | Singapore 2007-2009 | BSI | E. coli,  Klebsiella sp.  P. aeruginosa | 675 | 11.4% | Total hospitalization Cost in USD | Ref 1 | Coefficient -0.28  (-0.48, -0.09) | 0.005 |
| Yang 2010[66] | Taiwan 2006-2008 | BSI | E. coli, K. pneumoniae | 58 (12 ESBL+ and 46 ESBL-) | 75% ESBL+  0% ESBL- | Mean antibiotic cost ± SD | US$615  ± 424 | US$253  ± 269 | 0.01 |
| Sturkenboom 2005[64] | Netherlands; 1995-1999 | IAI | B. lactamase | 175 | 16% | Mean total cost | US$8118 | NR | NA |
| Kollef 2008[25] | US  2002-2006 | BSI | PARGNB | 76 | 78% | Mean total hospital cost  ± SD | $86,644  ± 64,433 | $68,597 ± 55,466 | 0.390 |
| Tumbarello 2010[65] | Italy  2006 | BSI | E. coli | 134 (37 ESBL+ and 97 ESBL-) | 54% ESBL+  7% ESBL-  20.1% | Mean total cost ± SD | US$17310.72 ± 3340.80 | US$11777.28 ± 1169 | 0.04 |
| **Indirect Evidence†** |  |  |  |  |  |  | **Result**  **Group 1** | **Result**  **Comparison** |  |
| Apisarnthanarak 2008[54] | Thailand 2003-2007 | BSI | E. coli  K. pneumoniae | 144 (36 ESBL+ and 108 ESBL-) | 56% ESBL+  7% ESBL- | Median total cost (IQR) | Resistant  US$615 (US$43- US$3173) | Susceptible:  US$214 (US$53- US$1861) | <0.001 |
| Lautenbach 2001[57] | US  1997-1998 | Pneumonia | E. coli  K. pneumoniae | 99 (33 ESBL+ and 66 ESBL-) | 52% ESBL+  NR ESBL- | Median hospital charges | Resistant:  US$66 590  (1.7 times higher 95% CI 1.01-2.88)* | Susceptible  US$22 231 | <0.001 |
| Lee 2007[58] | Taiwan 1996-2001 | Nosocomial BSI | A. baumannii | 92 | 15.2% MDR  4.3% non-MDR | Hospitalization cost ± SD | MDR  US$9348 ± 6323 | Non-MDR  US$4865 ± 4015 | 0.001 |
|  |  |  |  |  |  | Antibiotic therapy cost ± SD | US$2257 ± 1361 | US$1610 ± 1315 | 0.014 |
| MacVane 2014[60] | US  2011-2012 | UTI | E. coli  K. pneumoniae | 110  (55 ESBL+  55 ESBL-) | 76% ESBL+  1.8% ESBL- | Hospitalization cost (IQR) | Resistant:  US$10,741 (6846–15,819) | Susceptible: US$7,083 (5667–11,652) | 0.02 |
| Schwaber 2006[62] | Israel  2000-2003 | Bacteremia | E. coli, Klebsiella sp., Proteus sp. | 198  (99 ESBL+  99 ESBL-) | 66% ESBL+  7% ESBL- | Mean total cost per patient | Resistant:  US$46 970 (65 509 shekels) | Susceptible: US$16 877 (23 538 shekels) | <0.001 |

BSI, Blood stream infection; CI, Confidence Interval; ESBL, Extended spectrum beta-lactamase; ICU, Intensive care unit; LOS, Length of stay; MDR, Multi-drug resistant; NR, Not reported; OR, Odds Ratio, PARGNB, P. aeruginosa, Acinetobacter, and Stenotrophomonas maltophilia; SD, Standard deviation; UTI, Urinary Tract Infection; VAP, Ventilator-associated pneumonia

* Controlled for APACHE score in multivariate analysis

† Resistant group received more inappropriate therapy than the susceptible group

**Table 2. Study characteristics and results of length of stay**

| **Author**  **Year** | **Country Recruited Yr** | **Infection site** | **Pathogen (sp.)** | **Total N** | **Receiving IAT** | **Type of Stay** | **Result IAT** | **Result AAT** | | **P-value** |
| --- | --- | --- | --- | --- | --- | --- | --- | --- | --- | --- |
| Bailey 2013[55] | USA  2009-2011 | UTI | E. coli | 222 | 82% | Hospital LOS | OR 1.22  95% CI 0.59, 2.53 | | Ref:1 | 0.59 |
| Bare 2006[56] | Spain  1998-2000 | IAI | NR | 376 | 14% | LOS | 21.35±31.34 | | 18.22±22.6 | 0.36 |
| Falagas 2006[17] | Greece  2002-2004 | BSI | Acinetobacter baumannii | 40 | 55% | ICU LOS | 21.3±22.9 | | 24.4±18.2 | 0.46 |
| Joung 2010[22] | South Korea 2000-2006 | VAP | Acinetobacter baumannii | 116 | 66% | LOS | 45.5±76.8 | | 46.0±45.6 | 0.97 |
| Lee 2011[59] | South Korea 2000-2004 | UTI | E. coli | 164 | 17.7% | LOS | 13.3±14.0 | | 8.7±6.8 | 0.002 |
|  |  |  |  |  |  | ICU LOS | 0.2±0.8 | | 0.4±1.6 | 0.61 |
| Lye 2012 Ng 2012[31,38] | Singapore  2007-2009 | BSI | Enterobacteriaceae Klebsiella sp. P. aerugionosa; Acinetobacter sp. | 675 | 88.6% | LOS | NR | | NR | 0.46 |
| Shorr 2011[63] | USA  2002-2007 | BSI | Enterobacteriaceae, Klebsiella; P. aerugionosa; Acinetobacter; Enterobacter sp.; Proteus | 760 | 68.7% | LOS | 11 | | 8 | 0.028 |
| Thom 2008  Osih 2007[45,46,61] | USA  2001-2005 | BSI | E. coli  Klebsiella sp. | 416 | 26.7% | LOS | NR | | NR | 0.24 |

AAT, Appropriate initial antibiotic therapy; BSI, Blood Stream Infection; CI, Confidence Interval; ICU, Intensive Care Unit; IAI, Intra-abdominal infection; IAT, Inappropriate initial antibiotic therapy; LOS, Length of stay; N, number; NR, Not reported; OR, Odds Ratio; UTI, Urinary Tract Infection; VAP, Ventilator-associated pneumonia
